# Determinants of postnatal care utilization and maternal practices in rural areas of Lao People’s Democratic Republic: a community-based cross-sectional study

**DOI:** 10.1186/s12884-025-07798-4

**Published:** 2025-07-02

**Authors:** Souphaphone Sadettan, Souphalak Inthaphatha, Vanpheng Phanthanalay, Douangta Leungmylay, Sengthong Seumlamvanh, Bounfeng Phoummalaysith, Kimihiro Nishino, Eiko Yamamoto

**Affiliations:** 1 Department of Healthcare Administration, Nagoya University Graduate School of Medicine, 65 Tsurumai-cho, Showa-ku, Nagoya, 466-8550 Japan; 2 Cabinet, Ministry of Health, Vientiane Capital, Lao People’s Democratic Republic; 3 Asian Satellite Campuses Institute, Nagoya University, Nagoya, Japan; 4 Vientiane Provincial Health Department, Phonhong, Lao People’s Democratic Republic; 5 Bolikhamsai Provincial Health Department, Pakxan, Lao People’s Democratic Republic; 6 Khammouane Provincial Health Department, Thakhek, Lao People’s Democratic Republic; 7 Ministry of Health, Vientiane Capital, Lao People’s Democratic Republic

**Keywords:** Lao PDR, Maternal and child health, Postnatal care, Postpartum women

## Abstract

**Background:**

Postnatal care (PNC) contributes to reducing maternal and newborn mortality and morbidity. This study aims to identify the utilization of PNC and factors associated with receiving at least two PNC services for mothers and babies in Lao People’s Democratic Republic (Lao PDR).

**Methods:**

A cross-sectional study was conducted involving 533 women at 6–12 weeks postpartum in Vientiane, Bolikhamsai, and Khammouane Provinces from October to December 2022. Socio-demographic factors, obstetrics and infant factors, attitudes towards visiting health facilities and PNC, and knowledge, attitudes, and practice of maternal and child health were collected. The utilization of PNC for women and for babies was analyzed. Logistic regression analyses were performed on having two PNC services or more for mothers and babies.

**Results:**

Most mothers were 19–29 years old, had education at secondary school, had four or more antennal care visits, and had childbirth at health facilities. The coverage of PNC within the 24 h after birth (PNC 1) for mothers was 77.9% and PNC 1 for newborns was 84.2%. The coverage of two or more PNC services for both mothers and their babies was 43.3%. More than 90% of the women had an educational session on breastfeeding and took iron supplements after their most recent childbirth, approximately 80% used appropriate contraception methods, and 62.7% completed vaccinations for their most recent babies according to the baby’s age. Approximately 10% of women reported that factors such as income, distance, and transportation serve as barriers to accessing PNC. Factors associated with having two or more PNC services for mothers and babies were being single/divorced, having a monthly household income ≥ 1,000,000 Lao Kip (approximately 45 USD), living outside the city, and having the most recent childbirth at health facilities.

**Conclusions:**

While PNC 1 coverage was relatively high, continued PNC services remained low. Promoting institutional delivery with a minimum 24-hour stay postpartum may improve PNC uptake. The Lao government should consider reintroducing targeted financial support for postpartum women, especially for those with a low-income. Strengthening community outreach through local health workers and culturally appropriate education can further enhance access to and uptake of PNC.

## Introduction

Postnatal period refers to the six weeks just after childbirth and it is a critical period for mothers and babies because most maternal and infant deaths occur during the postnatal period, especially during the first week after childbirth [1–4]. A systematic review of maternal deaths during the postpartum period reported that 48.9% and 24.5% occurred on the first day and from the second to the seventh day [5]. The major cause of death was postpartum hemorrhage followed by embolism on the first day; and postpartum eclampsia followed by hypertensive disorders in the first week. Postnatal care (PNC) is a part of continuum care for women after childbirth and newborns and PNC services should be provided to detect and manage problems after childbirth, prevent diseases, and promote healthy practices. The World Health Organization (WHO) recommends that both mothers and babies should receive PNC at least four times, namely for the first 24 h, between 48 and 72 h, between the 7th and 14th day, and during the 6th week after birth [1, 6]. The most important care is physical assessment in the first 24 h, such as vaginal bleeding and uterine contraction of mothers and breathing, temperature, and no abnormal signs (jaundice and convulsions) of newborns. PNC services should also include nutrition interventions, screening and prevention of postpartum depression, provision of contraceptive information and services, vaccination service, infant growth and development, and promotion of breastfeeding. PNC services are necessary to prevent maternal and child deaths and to improve their health condition.

In most low- and middle-income countries (LMICs), the coverage of PNC is lower than that of antenatal care (ANC), skilled birth attendant, and institutional delivery, with the continuum of care most often interrupted at the PNC stage [7, 8]. However, PNC has the potential to have a greater positive effect than that of ANC and is similar to that of intrapartum care but at a lower estimated cost [9]. A systematic review estimated that PNC can reduce all-cause neonatal mortality by 10–50% [9]. Bang et al. reported that PNC at home by community health workers reduced neonatal mortality by 62% in rural areas of India [10]. There have been many studies on the utilization of PNC services for mothers and newborns in LMICs, especially in South Asia and Sub-Saharan Africa, and most studies used the data of the Demographic Health Surveys (DHS) and the Multiple Indicator Cluster Surveys (MICS). A systematic review of studies conducted in LMICs reported that having a richer wealth index, living in urban areas, having a higher education and well-paid jobs of mothers and their husbands, and having a shorter travelling time to PNC services were associated with having PNC for mothers and newborns [7, 11].

Lao People’s Democratic Republic (Lao PDR) is a lower middle-income country in Southeast Asia. The maternal mortality ratio (MMR, per 100,000 live births) decreased from 405 in 2005 to 126 in 2020 and the neonatal mortality ratio (NMR, per 1,000 live births) decreased from 33 in 2005 to 12 in 2023 [12–14]. The coverage of ANC (at least once) greatly improved from 26.7% in 2005 to 89.8% in 2023, and 71.6% of women had at least four ANC visits in 2023. The coverage of PNC was only 6.9% in 2005, but increased to 64.2% among mothers and 64.0% among newborns in 2023 [13]. However, only 32.6% of mothers received at least two postnatal signal care functions within two days after delivery. In Lao PDR, approximately 20% of women delivered outside health facilities and 14.6% of women who had childbirth at health facilities stayed in the health facilities less than 12 h [13]. Therefore, PNC is one of the critical steps to reducing MMR and NMR. MICS surveys (Lao Social Indicator Survey [LSIS]) were conducted in Lao PDR in 2011–2012, 2017, and 2023, but the timings of PNC services in the MICS data were “before leaving health facilities or before skilled birth attendant leaving home”, “within the first two days”, and “after the first week”, which were different from those in the WHO recommendations. Most previous studies using the data of DHS and MICS in LMICs also did not show the distribution of PNC services that were utilized by mothers and newborns. This study aimed to examine the distribution of maternal and newborn PNC utilization, identify factors associated with PNC utilization, and understand maternal and child health (MCH) knowledge and practices among Lao women.

## Materials and methods

### Study design and data collection

This is a cross-sectional study which was conducted in Vientiane, Bolikhamsai, and Khammouane Provinces in Lao PDR. The inclusion criteria of the study subjects were Lao women who had childbirth 6–12 weeks before the data collection, who lived in the three provinces, who could communicate in Lao and/or Hmong languages, and who agreed to participate in this study. Written informed consent was obtained from each participant. When a woman was younger than 18 years old, written informed consent was obtained from her legal representative who was 18 years old or older.

Data were collected from 600 postpartum women through a face-to-face interview using a questionnaire from October 3 to December 30, 2022. In each province, 200 women who had delivered between August and October 2022 were randomly selected using a lottery method. The total number of deliveries from August to October in 2022 was 2,072 in Vientiane Province, 1,746 in Bolikhamsai Province, and 1,907 in Khammouane Province. The sample size was estimated to be 544 based on a prevalence of 50%, with a 95% confidence interval and a margin of error less than 4%. Each data collection team consisted of an obstetrics and gynecological doctor, a nurse, and a midwife who worked for the provincial hospital, district hospitals, and health centers in each province. The teams visited the homes of the selected 200 women in their respective provinces. Finally, 533 women were included in the study after 67 women were excluded because they were 4–5 weeks postpartum.

A structured questionnaire was developed based on previous studies on maternal and newborn care in LMICs by the authors to collect data for this study [8, 9, 11, 15–21]. The questionnaire included (i) socio-demographic data, (ii) obstetrics and infant data, (iii) attitudes towards visiting health facilities and PNC, and (iv) knowledge, attitudes, and practice of MCH. It was reviewed by three maternal health experts for content validity and translated to the Lao language, with back-translation performed to ensure accuracy. The main researcher of the study provided guidance and training on data collection to the team members in April 2022. The questionnaire was revised after a pre-test was performed including 100 postpartum women from April 2022 to July 2022.

### Socio-demographic data

Social-demographic data included age, residence, marital status, educational level, occupation, ethnolinguistic group, monthly household income, partner’s age, partner’s educational level, and partner’s ethnolinguistic group. Age was categorized into ≤ 18 years old, 19–29 years old, and ≥ 30 years old. Residence was divided into ‘in the city’ and ‘outside the city’. Marital status had four choices, which were divided into two groups, such as married/cohabiting and single/divorced. The educational level of women and their partners was categorized into four groups namely none/primary school, secondary school, high school, and college/university. Women’s occupations were categorized as housewife, merchant, government/non-governmental organization (NGO)/private company employee, and other (student, farmer, and factory worker). Ethnolinguistic groups included Lao-Tai, Hmong-Nien, Mon-Khmer, and other. Monthly household income was categorized into < 1,000,000 Lao Kip (LAK) (approximately 45 USD), 1,000,000–2,500,000 LAK, and > 2,500,000 LAK (1 USD = 22,240 LAK as of September 11, 2024).

### Obstetrics and infant data

Obstetrics data included parity, gravida, history of abortions, data on the most recent pregnancy and childbirth (intent of the pregnancy, number of ANC visits and women’s complication during the pregnancy, place and mode of delivery, birth attendant, gestational week at birth, and birth weight), and data on the last child (age of the last child at the interview, baby’s complication after birth, and PNC services). Parity and gravida were divided into 1–2 and 3 or more. Abortions included spontaneous and induced abortions. Number of ANC visits was categorized into 0–3 and 4 or more. Place of delivery was divided into health facilities (hospitals and health centers) and home/others. Mode of delivery was categorized into vaginal delivery and cesarean session. Responses about birth attendant were categorized by skilled birth attendant; ‘yes’ (doctor, nurse, and midwife) and ‘no’ (traditional birth attendant, family member, and no one). Gestational age was determined based on the first day of the last menstrual period, or, if this was unknown, estimated using uterine length and abdominal circumference [22]. Gestational week at birth was categorized into < 37 weeks and ≥ 37 weeks according to the definition of preterm. Birth weight was divided into < 2,500 g and ≥ 2,500 g based on the definition of low birth weight. Age of the last baby was divided into 6–9 weeks and 10–12 weeks. Women were asked if their last baby had complications after birth and they answered ‘yes’ or ‘no’. PNC for mothers includes checking physical recovery, breastfeeding support, family planning support, health education, and management of complications arising from pregnancy or delivery [1, 22]. PNC for babies encompasses all medical, developmental, and supportive interventions, such as immediate care after birth, physical health and growing check, breastfeeding support, immunization, hygiene and cord care, and education to caregivers. Regarding PNC, women answered if they received PNC for mothers or for babies during the first 24 hours after birth (PNC 1), between 48 and 72 hours (PNC 2), between the 7th and 14th day (PNC 3), and in the 6th week after birth (PNC 4). When a mother and her newborn receive PNC from the same provider, at the same location, and during the same visit, it was counted as one PNC visit for the mother and one PNC visit for the newborn.

### Attitudes towards visiting health facilities and PNC

The participants were asked “Who decides when you go to health facilities?” and selected one from three choices (‘myself’, ‘partner’, and ‘mother/mother-in-law’). Next, they were asked if they had confidence in their access to health facilities and the choices were ‘yes’ or ‘no’. To understand the barriers to PNC for Lao mothers, the following questions were used: “Does your income prevent you from receiving PNC?”, “Does distance from health facilities prevent you from receiving PNC?”, and “Do transportation difficulties prevent you from receiving PNC?”

### Education and practice of MCH

The aims of PNC include providing education about MCH and supplements to mothers. The participants were asked if they had educational sessions of breastfeeding, nutrition, and illness after the last childbirth and where they had received them (provincial hospital, district hospital, health center, and community). They were asked if they had iron, vitamin B1, and folinic acid after the last childbirth. Regarding breastfeeding, the participants were asked if they gave their babies only breastmilk, only formula, or both breastmilk and formula, and if they gave something other than breastmilk or formula. They were also asked how long they wanted to give breastmilk and selected one from ‘until 3 months old’, ‘until 6 months old’, and ‘until 6 months old or later.’ Contraception methods included pills, injection, condom, implants, female sterilization, and others. The place of receiving contraception was categorized into hospitals/health centers, pharmacies, and others. The participants were asked if their last child completed the immunizations that should have been completed by their age.

### Statistical analysis

The Lao government has set a target of achieving 70% coverage of at least one PNC visit within the first 48 h after birth by 2025. However, to better align with the WHO recommendation of at least four PNC contacts within six weeks postpartum, this study defined the outcome as receiving two or more PNC services for both mothers and babies, based on the mean number of PNC services: 1.52 for mothers and 1.61 for babies. When mothers had two or more PNC services for mothers but no or one PNC services for babies, they were categorized into the other group. To identify the factors associated with having two or more PNC services, we performed multivariable logistic regression analysis using the forced-entry method (Model 1). Independent variables were age, residence, marital status, educational level, occupation, ethnolinguistic group, monthly household income, partner’s age, partner’s educational level, partner’s ethnolinguistic group, parity, history of abortions, intent of the last pregnancy, number of ANC visits during the last pregnancy, place and mode of the last delivery, birth attendant at the last childbirth, gestational week and birth weight of the last child, and “Who decides when you go to health facilities?” Additionally, we performed stepwise forward-selection (Model 2) and backward-selection (Model 3) analyses as supplementary approaches to explore the consistency of variable selection and effect estimates. Variables were added when the *P*-value was less than 0.05 and removed when the *P*-value was greater than 0.1. A correlation matrix was created to confirm that there was no strong correlation (γ > 0.80) between the independent variables. The result of the Hosmer-Lemeshow test of each model was confirmed to be *P* > 0.05. Statistical analysis was performed using the Statistical Package for Social Sciences, version 25 (IBM SPSS Inc.). Statistical significance was set at *P* < 0.05.

### Ethical issues

This study was approved by the National Ethics Committee for Health Research in Lao PDR (033/NECHR). Written informed consent was obtained from each participant before data collection.

## Results

### Utilization of PNC 1–4 for mothers and babies

A total of 533 women who lived in Vientiane, Bolikhamsai, and Khammouane Provinces and who were at 6–12 weeks postpartum were included in this study. Regarding PNC for mothers, 415 women (77.9%) received PNC 1 and 295 women (55.3%) received PNC 4 (Table 1). Almost the same percentage of babies received PNC 1 (84.2%) and PNC 4 (54.6%) as their mothers. However, only 63 mothers (11.8%) and 82 babies (15.4%) received PNC 2 and 38 mothers (7.1%) and 36 babies (6.8%) received PNC 3. There were 38 mothers (7.1%) and 23 babies (4.3%) who had no PNC, and 19 pairs of mother and baby had no PNC.

**Table 1:** Number and percentage of participants who had postnatal care services for mothers and babies (*N* = 533)

		PNC for mothers					
		None	PNC 1 (in 24 h)	PNC 2 (48–72 h)	PNC 3 (7–14 days)	PNC 4 (6th week)	Total
PNC for babies	None	19 (3.6)	3 (0.6)	0 (0.0)	0 (0.0)	1 (0.2)	23 (4.3)
	PNC 1 (in 24 h)	10 (1.9)	407 (76.4)	54 (10.1)	28 (5.3)	254 (47.7)	449 (84.2)
	PNC 2 (48–72 h)	1 (0.2)	64 (12.0)	56 (10.5)	10 (1.9)	66 (12.4)	82 (15.4)
	PNC 3 (7–14 days)	1 (0.2)	23 (4.3)	4 (0.8)	25 (4.7)	19 (3.6)	36 (6.8)
	PNC 4 (6th week)	11 (2.1)	216 (40.5)	50 (9.4)	16 (3.0)	260 (48.8)	291 (54.6)
Total		38 (7.1)	415 (77.9)	63 (11.8)	38 (7.1)	295 (55.3)	533 (100)

Values are presented as number of participants (%)

PNC, postnatal care

### Total number of PNC services for mothers and babies

Regarding the total number of PNC services, most mothers had one PNC service (*n* = 234, 43.9%) followed by two services (*n* = 207, 38.8%) and most babies had one service (*n* = 237, 44.5%) followed by two services (*n* = 199, 37.3%) (Table 2). The coverage of at least one PNC service was 92.9% among mothers and 95.7% among babies. There were 231 pairs (43.3%) of mothers and babies who had two or more PNC services. When the combination of the number of PNC services for mothers and babies was examined, the majority was receiving one PNC service for mothers and one PNC service for babies (*n* = 191, 35.8%) followed by having two PNC services for mothers and two PNC services for babies (*n* = 167, 31.3%). There was only one mother and one baby who had four PNC services but no pair of mother and baby who had four PNC services.

**Table 2:** Number and percentage of participants according to the total number of PNC services for mothers and babies (*N* = 533)

		Total number of PNC services for mothers					Total
		0	1	2	3	4	
Total number of PNC services for babies	0	19 (3.6)	4 (0.8)	0 (0.0)	0 (0.0)	0 (0.0)	23 (4.3)
	1	16 (3.0)	191 (35.8)	30 (5.6)	0 (0.0)	0 (0.0)	237 (44.5)
	2	2 (0.4)	27 (5.1)	167 (31.3)	3 (0.6)	0 (0.0)	199 (37.3)
	3	1 (0.2)	12 (2.3)	10 (1.9)	49 (9.2)	1 (0.2)	73 (13.7)
	4	0 (0.0)	0 (0.0)	0 (0.0)	1 (0.2)	0 (0.0)	1 (0.2)
Total		38 (7.1)	234 (43.9)	207 (38.8)	53 (9.9)	1 (0.2)	533 (100)

Values are presented as number of participants (%)

PNC, postnatal care; N, number

### Social-demographic factors of the study participants

The number of participants were 189 in Vientiane Province, 168 in Bolikhamsai Province, and 176 in Khammouane Province (Table 3). Most women were 19–29 years old (56.7%), lived outside the city (88.0%), were married or cohabiting (96.6%), and had lived with their partners for five years or longer (41.3%), had secondary education (30.8%), were housewives (59.8%), belonged to Lao-Tai ethnicity (71.5%), and had a monthly household income of 1,000,000–2,500,000 LAK (49.2%). Partners of most women were 30 years old or older (55.0%), had education at high school (29.8%), and belonged to Lao-Tai ethnicity (70.5%).

**Table 3:** Social-demographic factors of postpartum women and their partners (*N* = 533)

Variables	Total (*N* = 533)		2 or more PNC services for mothers and babies (*N* = 231)	
	*N* (%)		*n*	% (95% CI)
**Province**				
Vientiane	189 (35.5)	86		45.5 (39.0–52.0)
Bolikhamsai	168 (31.5)	62		36.9 (31.3–42.5)
Khammouane	176 (33.0)	83		47.2 (40.2–54.1)
**Age (years old)**				
≤ 18	52 (9.8)	15		28.8 (21.0–36.7)
19–29	302 (56.7)	134		44.4 (39.4–49.4)
≥ 30	179 (33.6)	82		45.8 (39.1–52.5)
**Residence**				
Outside the city	469 (88.0)	207		44.1 (40.1–48.1)
In the city	64 (12.0)	24		37.5 (28.3–46.7)
**Marital status**				
Married/cohabiting	515 (96.6)	220		42.7 (39.0-46.4)
Single/divorced	18 (3.4)	11		61.1 (32.9–89.3)
**Relationship duration**				
< 2 years	140 (26.3)	43		30.7 (25.6–35.8)
2–< 5 years	173 (32.5)	88		50.9 (43.3–58.4)
≥ 5 years	220 (41.3)	100		45.5 (39.4–51.5)
**Education**				
None/primary	149 (28.0)	43		28.9 (24.2–33.5)
Secondary	164 (30.8)	72		43.9 (37.2–50.6)
High school	131 (24.6)	75		57.3 (47.4–67.1)
College/university	89 (16.7)	41		46.1 (36.5–55.6)
**Occupation**				
Housewife	319 (59.8)	139		43.6 (38.8–48.4)
Merchant	86 (16.1)	28		32.6 (25.7–39.4)
Government/private company/ NGO employee	98 (8.4)	48		49.0 (39.3–58.7)
Other^a^	30 (5.6)	16		53.3 (34.2–72.4)
**Ethnolinguistic group**				
Lao-Tai	381 (71.5)	181		47.5 (42.7–52.3)
Hmong-Mien	84 (15.8)	28		33.3 (26.2–40.5)
Mon-Khmer	36 (6.8)	9		25.0 (16.8–33.2)
Other	32 (6.0)	13		40.6 (26.5–54.7)
**Monthly household income (LAK)**				
< 1,000,000	103 (19.3)	23		22.3 (18.0-26.6)
1,000,000–2,500,000	262 (49.2)	119		45.4 (39.9–50.9)
> 2,500,000	168 (31.5)	89		53.0 (45.0–61.0)
**Partner’s age (years old)**				
≤ 18	14 (2.6)	2		14.3 (6.8–21.8)
19–29	226 (42.4)	100		44.2 (38.5–50.0)
≥ 30	293 (55.0)	129		44.0 (39.0-49.1)
**Partner’s education**				
None/primary	101 (19.0)	29		28.7 (23.1–34.3)
Secondary	146 (27.4)	52		35.6 (29.8–41.4)
High school	159 (29.8)	87		54.7 (46.2–63.2)
College/university	127 (23.8)	63		49.6 (41.0-58.2)
**Partner’s ethnolinguistic group**				
Lao-Tai	376 (70.5)	180		47.9 (43.0-52.7)
Hmong-Mien	85 (15.9)	28		32.9 (25.9–39.9)
Mon-Khmer	43 (8.1)	12		27.9 (19.6–36.2)
Other	29 (5.4)	11		37.9 (24.1–51.7)

NGO, non-governmental organization; LAK, Lao Kip; PNC, postnatal care

^a^Other included students, farmers, and factory workers

### Obstetrics and infant factors of the study participants

Of all the 533 women, 73.9% had 1–2 childbirths, 85.7% had no history of abortions, and 66.8% answered that the last pregnancy was an intended pregnancy (Table 4). Regarding the last pregnancy, 85.2% of the women had four ANC visits or more, 94.9% had childbirth at health facilities, 89.5% had vaginal delivery, and 94.7% had a skilled birth assistant. Twelve mothers (2.3%) had complications during the last pregnancy and six babies (1.1%) had complications after birth. At the time of data collection, the last babies of 287 women (53.8%) and 246 women (46.2%) were 6–9 weeks old and 10–12 weeks old, respectively.

**Table 4:** Obstetrics and infant factors of postpartum women (*N* = 533)

Variables	Total (*N* = 533)	2 or more PNC services for mothers and babies (*N* = 231)	
	*N* (%)	*n*	% (95%CI)
**Parity**			
1–2	394 (73.9)	181	45.9 (41.4–50.5)
≥ 3	139 (26.1)	50	36.0 (30.0–42.0)
**Gravida**			
1–2	367 (68.9)	169	46.0 (41.3–50.8)
≥ 3	166 (31.1)	62	37.3 (31.7–43.0)
**History of abortions**			
No	457 (85.7)	199	43.5 (39.6–47.5)
Yes	76 (14.3)	32	42.1 (32.6–51.6)
**Intended pregnancy (the last pregnancy)**			
Yes	356 (66.8)	159	44.7 (40.0–49.3)
No	177 (33.2)	72	40.7 (34.7–46.7)
**ANC visits during the last pregnancy**			
0–3 times	79 (14.8)	22	27.8 (21.7–34.0)
≥ 4 times	454 (85.2)	209	46.0 (41.8–50.3)
**Complication during the last pregnancy**			
No	521 (97.7)	223	42.8 (39.1–46.5)
Yes	12 (2.3)	8	66.7 (28.9–104.4)
**Place of the last childbirth**			
Health facility	506 (94.9)	228	45.1 (41.1–49.0)
Home and others	27 (5.1)	3	11.1 (6.9–15.3)
**Mode of delivery (the last childbirth)**			
Vaginal delivery	477 (89.5)	199	41.7 (38.0–45.5)
Caesarean section	56 (10.5)	32	57.1 (42.2–72.1)
**Skilled birth attendant (the last childbirth)**			
Yes	505 (94.7)	227	45.0 (41.0–48.9)
No	28 (5.3)	4	14.3 (9.0–19.6)
**Gestational week at birth of the last baby (weeks)**			
< 37	60 (11.3)	18	30.0 (22.4–37.6)
≥ 37	473 (88.7)	213	45.0 (41.0–49.1)
**Birth weight of the last baby (g)**			
< 2,500	33 (6.2)	14	42.4 (27.9–56.9)
≥ 2,500	500 (93.8)	217	43.4 (39.6–47.2)
**Age of the last child at the interview (weeks old)**			
6–9	287 (53.8)	122	42.5 (37.6–47.4)
10–12	246 (46.2)	109	44.3 (38.8–49.8)
**Complication of the last baby after birth**			
No	527 (98.9)	227	43.1 (39.4–46.8)
Yes	6 (1.1)	4	66.7 (13.3–120.0)

ANC, antenatal care; PNC, postnatal care

### Attitudes toward visiting health facilities and receiving PNC

Regarding the attitudes towards visiting health facilities and receiving PNC, 55.2% of the women answered that they decide when they go to health facilities, but 26.6% and 18.2%, respectively, answered that their partners and mothers or mothers-in-law decide (Table 5). Almost all women (95.7%) have confidence in their access to health facilities, but approximately 10% of the women answered income (13.3%), distance from healthcare facilities (9.9%), and transportation difficulties (15.6%) prevent them from receiving PNC.

**Table 5:** Attitudes toward visiting health facilities and receiving postnatal care among postpartum women (*N* = 533)

Variables	Total (*N* = 533)	2 or more PNC services for mothers and babies (*N* = 231)	
	*N* (%)	*n*	% (95% CI)
**Who decides when you go to health facilities?**			
Myself	294 (55.2)	136	46.3 (41.0-51.5)
Partner	142 (26.6)	61	43.0 (35.9–50.0)
Mother/mother-in-law	97 (18.2)	34	35.1 (28.1–42.0)
**Do you have confidence in your access to health facilities?**			
Yes	510 (95.7)	222	43.5 (39.8–47.3)
No	23 (4.3)	9	39.1 (23.1–55.1)
**Does your income prevent from you receiving PNC?**			
No	462 (86.7)	223	48.3 (43.9–52.3)
Yes	71 (13.3)	8	11.3 (8.6–13.9)
**Does distance from healthcare facilities prevent from you receiving PNC?**			
No	480 (90.1)	227	47.3 (43.1–51.5)
Yes	53 (9.9)	4	7.5 (5.5–9.6)
**Do transportation difficulties prevent you from receiving PNC?**			
No	450 (84.4)	221	49.1 (44.6–53.6)
Yes	83 (15.6)	10	12.0 (9.5–14.6)

PNC, postnatal care

### Factors associated with having two or more PNC services for mothers and babies

Binary logistic regression analysis showed factors associated with having two or more PNC for mothers and babies were being 19–29 years old (OR = 1.97, 95% CI 1.04–3.74) and 30 years old or older (OR = 2.09, 95% CI 1.07–4.07) than 18 years or younger; having education at secondary school (OR = 1.93, 95% CI 1.21–3.09), high school (OR = 3.30, 95% CI 2.01–5.42), and college or university (OR = 2.11, 95% CI 1.22–3.64) than no education; having a monthly household income of 1,000,000–2,500,000 LAK (OR = 2.89, 95% CI 1.71–4.89) and > 2,500,000 LAK (OR = 3.92, 95% CI 2.25–6.82) than < 1,000,000 LAK; partner’s age of 19–29 years old (OR = 4.76, 95% CI 1.04–21.77) and 30 years old or older (OR = 4.72, 95% CI 1.04–21.46) than 18 years or younger; partner’s education at high school (OR = 3.00, 95% CI 1.76–5.11) and college or university (OR = 2.44, 95% CI 1.40–4.25) than no education; having four ANC visits or more (OR = 2.21, 95% CI 1.31–3.74) than 0–3 ANC visits; and having caesarean section (OR = 1.86, 95% CI 1.06–3.26) than vaginal delivery (Table 6). Women’s ethnolinguistic group of Hmong-Mien (OR = 0.55, 95% CI 0.34–0.91) and Mon-Khmer (OR = 0.37, 95% CI 0.17–0.80) compared to Lao-Thai, partner’s ethnolinguistic group of Hmong-Mien (OR = 0.53, 95% CI 0.33–0.88) and Mon-Khmer (OR = 0.42, 95% CI 0.21–0.85) compared to Lao-Thai, having three children or more (OR = 0.66, 95% CI 0.48–0.99) than 1–2 children, having a history of abortions (OR = 0.48, 95%CI 0.28–0.82), having the last childbirth outside health facilities (OR = 0.15, 95% CI 0.05–0.51), having no skilled birth attendant (OR = 0.20, 95% CI 0.07–0.60), and having premature delivery (OR = 0.52, 95% CI 0.29–0.94) were negatively associated with having two or more PNC for mothers and babies.

**Table 6:** Logistic regression analysis on having two or more postnatal care services for mothers and babies

Variables	Unadjusted		Adjusted^b^					
			Model 1		Model 2		Model 3	
	OR (95% CI)	*P*	AOR (95% CI)	*P*	AOR (95% CI)	*P*	AOR (95% CI)	*P*
**Age (years old)**								
≤ 18	1 (Reference)		1 (Reference)		-		-	
19–29	1.97 (1.04–3.74)	0.039	1.40 (0.59–3.31)	0.447	-		-	
≥ 30	2.09 (1.07–4.07)	0.031	1.72 (0.65–4.57)	0.278	-		-	
**Residence**								
Outside the city	1 (Reference)		1 (Reference)		1 (Reference)		1 (Reference)	
In the city	0.76 (0.44–1.30)	0.316	0.41 (0.22–0.77)	0.006	0.53 (0.30–0.95)	0.032	0.46 (0.25–0.83)	0.010
**Marital status**								
Married/cohabiting	1 (Reference)		1 (Reference)		1 (Reference)		1 (Reference)	
Single/divorced	2.11 (0.80–5.52)	0.130	14.66 (2.93–73.36)	0.001	8.23 (2.08–32.58)	0.003	8.65 (2.12–35.35)	0.003
**Education**								
None/primary	1 (Reference)		1 (Reference		1 (Reference)			
Secondary	1.93 (1.21–3.09)	0.006	1.28 (0.69–2.39)	0.434	1.77 (1.06–2.95)	0.028		
High school	3.30 (2.01–5.42)	< 0.001	1.54 (0.76–3.13)	0.229	2.84 (1.65–4.88)	< 0.001		
College/university	2.11 (1.22–3.64)	0.008	0.94 (0.38–2.36)	0.897	1.87 (1.00-3.48)	0.049		
**Occupation**								
Housewife	1 (Reference)		1 (Reference)		-		-	
Merchant	0.63 (0.38–1.03)	0.067	1.12 (0.61–2.04)	0.716	-		-	
Government/private company/NGO employee	1.24 (0.79–1.96)	0.347	0.85 (0.47–1.52)	0.582	-		-	
Other^a^	1.48 (0.70–3.14)	0.306	1.30 (0.56–3.02)	0.547	-		-	
**Ethnolinguistic group**								
Lao-Tai	1 (Reference)		1 (Reference)		-		-	
Hmong-Mien	0.55 (0.34–0.91)	0.019	2.01 (0.35–11.43)	0.431	-		-	
Mon-Khmer	0.37 (0.17–0.80)	0.012	0.33 (0.05–2.38)	0.273	-		-	
Other	0.76 (0.36–1.57)	0.455	1.28 (0.34–4.83)	0.716	-		-	
**Monthly household income (LAK)**								
< 1,000,000	1 (Reference)		1 (Reference)		1 (Reference)		1 (Reference)	
1,000,000–2,500,000	2.89 (1.71–4.89)	< 0.001	2.09 (1.14–3.83)	0.017	2.44 (1.41–4.25)	0.002	2.16 (1.22–3.83)	0.008
> 2,500,000	3.92 (2.25–6.82)	< 0.001	2.77 (1.28–5.48)	0.004	3.07 (1.69–5.59)	< 0.001	2.70 (1.45–5.04)	0.002
**Partner’s age (years old)**								
≤ 18	1 (Reference)		1 (Reference)		-			
19–29	4.76 (1.04–21.77)	0.044	2.79 (0.47–16.54)	0.259	-			
≥ 30	4.72 (1.04–21.46)	0.045	2.01 (0.32–12.50)	0.455	-			
**Partner’s education**								
None/primary	1 (Reference)		1 (Reference)		-		1 (Reference)	
Secondary	1.37 (0.79–2.38)	0.257	1.11 (0.54–2.28)	0.775	-		1.36 (0.73–2.54)	0.335
High school	3.00 (1.76–5.11)	< 0.001	1.94 (0.91–4.15)	0.087	-		2.78 (1.50–5.17)	0.001
College/university	2.44 (1.40–4.25)	0.002	1.76 (0.74–4.18)	0.198	-		2.07 (1.07-4.00)	0.031
**Partner’s ethnolinguistic group**								
Lao-Tai	1 (Reference)		1 (Reference)					
Hmong-Mien	0.53 (0.33–0.88)	0.013	0.38 (0.07–2.15)	0.272	-		-	
Mon-Khmer	0.42 (0.21–0.85)	0.015	1.21 (0.24–6.24)	0.819	-		-	
Other	0.67 (0.31–1.45)	0.304	0.54 (0.13–2.22)	0.391	-		-	
**Parity**								
1–2	1 (Reference)		1 (Reference)		-		-	
≥ 3	0.66 (0.48–0.99)	0.042	1.14 (0.59–2.19)	0.695	-		-	
**Gravity**								
1–2	1 (Reference)		-		-		-	
≥ 3	0.70 (0.48–1.02)	0.061	-		-		-	
**History of abortions**								
No	1 (Reference)		1 (Reference)		-		-	
Yes	0.48 (0.28–0.82)	0.007	0.61 (0.28–1.36)	0.230	-		-	
**Intended pregnancy (the last pregnancy)**								
Yes	1 (Reference)		1 (Reference)		-		-	
No	0.85 (0.59–1.22)	0.382	0.85 (0.52–1.39)	0.525	-		-	
**ANC visits during the last pregnancy**								
0–3 times	1 (Reference)		1 (Reference)		-		-	
≥ 4 times	2.21 (1.31–3.74)	0.003	1.29 (0.67–2.49)	0.449	-		-	
**Mother’s complication during the last pregnancy**								
No	1 (Reference)		-		-		-	
Yes	2.67 (0.79–8.99)	0.112	-		-		-	
**Place of the last childbirth**								
Health facilities	1 (Reference)		1 (Reference)		1 (Reference)		1 (Reference)	
Home and others	0.15 (0.05–0.51)	0.002	0.11 (0.01–2.34)	0.157	0.11 (0.02–0.51)	0.005	0.11 (0.02–0.48)	0.004
**Mode of delivery (the last childbirth)**								
Vaginal delivery	1 (Reference)		1 (Reference)		-		1 (Reference)	
Caesarean section	1.86 (1.06–3.26)	0.029	1.93 (0.99–3.77)	0.052	-		1.95 (1.04–3.65)	0.037
**Skilled birth attendant (the last childbirth)**								
Yes	1 (Reference)		1 (Reference)		-		-	
No	0.20 (0.07–0.60)	0.004	1.49 (0.09–23.54)	0.776	-		-	
**Gestational week at birth of the last baby (weeks)**								
< 37	0.52 (0.29–0.94)	0.029	0.47 (0.24–0.93)	0.031	-		0.50 (0.27–0.92)	0.027
≥ 37	1 (Reference)		1 (Reference)		-		1 (Reference)	
**Birth weight of the last baby (g)**								
< 2,500	0.96 (0.47–1.96)	0.913	1.13 (0.47–2.70)	0.785	-		-	
≥ 2,500	1 (Reference)		1 (Reference)		-		-	
**Age of the last child at the interview (weeks old)**								
6–9	1 (Reference)		-		-		-	
10–12	1.08 (0.76–1.52)	0.676	-		-		-	
**Complication of the last baby after birth**								
No	1 (Reference)		-		-		-	
Yes	2.64 (0.48–14.56)	0.264	-		-		-	
**Who decides when you go to health facilities?**								
Myself	1 (Reference)		1 (Reference)		-		-	
Husband	0.87 (0.58–1.31)	0.516	1.10 (0.68–1.79)	0.693	-		-	
Mother/mother-in-law	0.63 (0.39–1.01)	0.055	0.60 (0.34–1.05)	0.072	-		-	

OR, odds ratio; CI, confidence interval; AOR, adjusted odds ratio; NGO, non-governmental organization; LAK, Lao Kip

Model 1, forced-entry (Hosmer-Lemeshow test: *P* = 0.057); Model 2, stepwise forward-selection (Hosmer-Lemeshow test: *P* = 0.592); Model 3, stepwise backward-selection (Hosmer-Lemeshow test: *P* = 0.121)

^a^Other included students, farmers, and factory workers

^b^Adjusted by age, residence, marital status, educational level, occupation, ethnolinguistic group, monthly household income, partner’s age, partner’s educational level, partner’s ethnolinguistic group, parity, history of abortions, intent of the last pregnancy, number of ANC visits during the last pregnancy, place and mode of the last delivery, birth attendant at the last childbirth, gestational week and birth weight of the last child, and “who decides when you go to health facilities?”

Multivariable logistic regression analyses were performed including 20 variables using three models. In Model 1 (forced-entry), living outside the city compared to living in the city (adjusted odds ratio [AOR] = 0.41, 95% CI 0.22–0.77), marital status of single or divorced (AOR = 14.66, 95% CI 2.93–73.36) compared to being married or cohabiting, monthly household income of 1,000,000–2,500,000 LAK (AOR = 2.09, 95% CI 1.14–3.83) and > 2,500,000 LAK (AOR = 2.77, 95% CI 1.28–5.48) compared to < 1,000,000 LAK, and having term delivery (AOR = 0.47, 95% CI 0.24–0.93) were associated with having two or more PNC (Table 6). In Model 2 (forward-selection), living outside the city (AOR = 0.53, 95% CI 0.30–0.95), marital status of single or divorced (AOR = 8.23, 95% CI 2.08–32.58), having education at secondary school (AOR = 1.77, 95% CI 1.06–2.95), high school (AOR = 2.84, 95% CI 1.65–4.88), and at college or university (AOR = 1.87, 95% CI 1.00-3.48), monthly household income of 1,000,000–2,500,000 LAK (AOR = 2.44, 95% CI 1.41–4.25) and > 2,500,000 LAK (AOR = 3.07, 95% CI 1.69–5.59), and having delivery at health facilities (AOR = 0.11, 95% CI 0.02–0.51) were the associated factors. In Model 3 (backward-selection), living outside the city (AOR = 0.46, 95% CI 0.25–0.83), marital status of single or divorced (AOR = 8.65, 95% CI 2.12–35.35), monthly household income of 1,000,000–2,500,000 LAK (AOR = 2.16, 95% CI 1.22–3.83) and > 2,500,000 LAK (AOR = 2.70, 95% CI 1.45–5.04), partner’s education at high school (AOR = 2.78, 95% CI 1.50–5.17) and at college/university (AOR = 2.07, 95% CI 1.07-4.00), having delivery at health facilities (AOR = 0.11, 95% CI 0.02–0.48), having caesarean section (AOR = 1.95, 95% CI 1.04–3.65), and having term delivery (AOR = 0.50, 95% CI 0.27–0.92) were associated with having two or more PNC.

### Education and practice of maternal and child health

Of the 533 women, 96.8% had educational sessions on breastfeeding, 72.6% on nutrition, and 58.0% on illness (Table 7). The location of the educational sessions was a provincial hospital for 56.9% of the women and a district hospital for 53.3%. Almost all women took iron (91.7%) and vitamin B1 (87.2%) supplements, but only 28.5% of the women took folinic acid. Regarding breastfeeding, 88.9% of the women gave only breastmilk to their last babies in the first few weeks, 87.8% had never given anything other than breastmilk or formula, and 71.3% wanted to breastfeed longer than six months. The major contraception method was pills (52.2%) followed by injection (17.6%), and 71.3% of the women received contraception methods at hospitals or health centers. Of all the 533 women, 37.3% answered that their last babies did not receive all vaccines according to their age. Women who had two or more PNC services for mothers and babies were significantly more likely to have educational sessions, take supplements, use contraception, and follow the vaccination schedule for their babies.

**Table 7:** Education and practice of maternal and child health

Variables	Total (*N* = 533)	2 or more PNC services for mothers and babies		*P*
		Yes (*N* = 231)	No (*N* = 302)	
	*N* (%)	*n* (%)	*n* (%)	
**Did you have educational sessions after the last delivery? (multiple answer)**				
Breastfeeding	516 (96.8)	229 (99.1)	287 (95.0)	0.011
Nutrition	387 (72.6)	197 (85.3)	190 (62.9)	< 0.001
Illness	309 (58.0)	153 (66.2)	156 (51.7)	0.001
**Where did you have the educational session? (multiple answer)**				
Provincial hospital	302 (56.9)	153 (66.2)	149 (49.7)	< 0.001
District hospital	284 (53.3)	136 (58.9)	148 (49.0)	0.028
Health center	145 (27.2)	77 (33.3)	68 (22.5)	0.006
In the community	118 (22.1)	70 (30.3)	48 (15.9)	< 0.001
**Did you take supplements after the last childbirth? (multiple answer)**				
Iron	489 (91.7)	226 (97.8)	263 (87.1)	< 0.001
Vitamin B1	465 (87.2)	219 (94.8)	246 (81.5)	< 0.001
Folinic acid	152 (28.5)	83 (35.9)	69 (22.8)	0.001
None	21 (3.9)	0 (0.0)	21 (7.0)	< 0.001
**What method did you use to feed the last baby in the first few weeks?**				
Only breastmilk	474 (88.9)	203 (87.9)	271 (89.7)	
Only formula	16 (3.0)	10 (4.3)	6 (2.0)	
Breastmilk and formula	43 (8.1)	18 (7.8)	25 (8.3)	
**Have you given anything other than breastmilk or formula to the last baby?**				1.000
Yes	65 (12.2)	28 (12.1)	37 (12.3)	
No	468 (87.8)	203 (87.9)	265 (87.7)	
**How long do you want to breastfeed the last baby?**				0.041
Until 3 months old	9 (1.7)	5 (2.2)	4 (1.3)	
Until 6 months old	144 (27.6)	50 (21.6)	96 (31.1)	
> 6 months old	380 (71.3)	176 (76.2)	204 (67.5)	
**Which contraception method are you using?**				0.001
Pills	278 (52.2)	132 (57.1)	146 (48.3)	
Injection	94 (17.6)	36 (15.6)	58 (19.2)	
Condom	15 (2.8)	11 (4.8)	4 (1.3)	
Implants	16 (3.0)	10 (4.3)	6 (2.0)	
Female sterilization	24 (4.5)	11 (4.8)	13 (4.3)	
Other^a^	106 (19.9)	31 (13.4)	75 (24.8)	
**Where did you get the contraception method?**				< 0.01
Hospital/health center	380 (71.3)	188 (81.4)	192 (63.6)	
Pharmacy	64 (12.0)	24 (10.4)	40 (13.2)	
Other	89 (16.7)	19 (8.2)	70 (23.2)	
**Completion of vaccination for the last baby according to the age**				0.004
Yes	334 (62.7)	161 (69.7)	173 (57.3)	
No	199 (37.3)	70 (30.3)	129 (42.7)	

PNC, postnatal care

^a^Other includes intrauterine device (*n* = 2), female condom (*n* = 2), rhythm method (*n* = 4), withdrawal (*n* = 4), and others (*n* = 94)

## Discussion

This study showed that the coverage of PNC 1 for mothers was 77.9% and PNC 1 for babies was 84.2% in the three provinces in Lao PDR, while the coverage of PNC 2 and 3 was low. The coverage of two or more PNC services for both mothers and babies was 43.3%. Factors associated with having two or more PNC services for mothers and babies were living outside the city, being single or divorced, having a higher household income, and having institutional delivery. Approximately 10% of women answered that income, distance, and transportation prevent women from receiving PNC. Women who had more PNC services were more likely to have educational sessions, take supplements, use contraception, and follow the vaccination schedule for their babies.

According to the LSIS, the coverage of PNC within the first two days among mothers and newborns in Lao PDR was 47.2% and 47.1% in 2015–2017 and 64.2% and 64.0% in 2021–2023 [13, 23]. The results of this study showed that the PNC coverage among mothers and newborns has increased in Lao PDR. The reason for an increase in the PNC coverage may be because the WHO revised its recommendation from two PNC postnatal checkups (within 2–3 days and at six weeks) to four in the first six weeks in 2013 and the Lao Ministry of Health established the guidelines of PNC in 2020 [6, 22]. The coverage of PNC 1 in this study was slightly higher than that in the LSIS 2023. It may be because the percentage of institutional delivery in this study was higher than that in the LSIS 2023 (78.2%). However, of the women who had institutional delivery in this study, 18.0% answered that they did not have PNC 1. This finding suggests that PNC is not consistently or automatically provided to women following delivery at health facilities in Lao PDR. In LSIS 2017, 52.7% of women with institutional delivery had postpartum stay for 1–2 days and 19.6% were discharged from health facilities within six hours after delivery [23]. Furthermore, all the women who had home delivery in this study did not have a skilled birth attendant or PNC 1. These results suggest that the risk of not receiving timely PNC affects not only women who deliver at home but also those who give birth in health facilities in Lao PDR. To increase the coverage of PNC, institutional delivery with at least 24-hour stay after birth should be promoted [24].

The analysis of the maternal and newborn PNC utilization of PNC 1–4 showed that the coverage of PNC among mothers and newborns was similar and most mothers and newborns received PNC 1 and PNC 4. This is reasonable because most mothers and babies have PNC 1 after childbirth at health facilities and PNC 4 for babies includes diphtheria-tetanus-pertussis-hepatitis B-Haemophilus influenzae type B and oral polio vaccinations [25, 26]. The reason why most mothers and babies did not receive PNC 2 and 3 may be because of the belief in Lao PDR that women should stay at home for 30–40 days after childbirth [27]. Most previous studies on PNC in LMICs examined having at least one PNC within the first two days or within the first week [16, 17, 20, 21, 28], although four PNC services was examined in Sri Lanka because the coverage was high and PNC services were provided not only at health facilities but also at home [18]. A randomized control study in the US suggests that the additional home visit at a 2-week postpartum visit to a routine visit at a 6-week postpartum decreased the number of emergency visits to health facilities, although the additional early visit did not increase the likelihood of the attendance of patients in a routine visit [29]. These results suggest that PNC services at home is recommended in Lao PDR to increase the coverage of PNC services and reduce maternal and child mortality and morbidity.

Factors associated with having two or more PNC services for mothers and babies included having a higher household income and living outside the city. Systematic reviews reported that women who had a higher socioeconomic status and lived in urban areas were significantly more likely to receive PNC services in LMICs [11, 30]. In this study, more women outside the city answered that distance and transportation to health facilities were obstacles to receiving PNC service. However, the coverage of PNC 1 and 4 was higher among women outside the city than those in the city. Therefore, they may remain at health facilities longer after childbirth compared to women in the city, potentially resulting in higher PNC 1 coverage and greater exposure to information about six-week vaccinations due to their extended stay [31]. Other possible explanations include NGO-led home visit programs for PNC in rural areas and stronger community ties that encourage women to utilize health services.

In Lao PDR, PNC services are provided free of charge under the National Health Insurance (NHI) scheme. However, postpartum women from low-income households may still hesitate to visit health facilities for PNC due to indirect costs such as transportation fees, loss of income, and medication expenses [25, 31]. The Free Maternal and Child Health (MCH) policy, introduced in 2011 to eliminate financial barriers to MCH services, covered not only the cost of ANC, delivery, PNC, treatment, and referrals, but also provided lump-sum payments for transportation and food, along with incentives to encourage service utilization [31–33]. In 2015, the Free MCH policy was integrated into the NHI scheme, and financial support beyond direct MCH services was discontinued. To improve PNC coverage, the Lao government should consider reintroducing targeted financial support—such as transport stipends or conditional cash transfers—for postpartum women, especially for those with a low-income. Strengthening community outreach through local health workers and culturally appropriate education can further enhance access and uptake.

In this study, women who were single or divorced were significantly more likely to have two or more PNC services compared to those who were married or cohabiting. Previous studies conducted in Sub-Sahara African countries reported that single/cohabiting women were unlikely to receive PNC compared to married women [19, 34, 35]. However, a systematic review of PNC utilization in LMICs, which mostly include studies in Sub-Sahara Africa or South Asia, showed no association between marital status and PNC utilization [11]. Most studies conducted in Southeast Asia did not include marital status as an independent variable or analyzed the data of married women [20, 36–38]. The reasons why marital status was not included in independent variables in many studies in Southeast Asian countries may be the low percentage of single and divorced mothers. An analysis of secondary data of 50,619 ever-married women in five Southeast Asian countries, including Lao PDR, showed no association between marital status and usage of MCH services including PNC [39]. A previous study in Lao PDR that analyzed the data of women in union found that those who were cohabiting were significantly more likely to receive skilled birth assistance and PNC compared to married women [40]. In the current study, more women who were single or divorced reported that access, income, distance, or transportation were not obstacles to receiving PNC compared with married/cohabiting women. These results suggest that the characteristics of unmarried women in Laos may differ from those in Sub-Saharan Africa, where misunderstandings and stigma toward unmarried women influence their access to PNC services.

This study has some limitations. First, there may be a recall bias because the study participants answered about their past experiences and practice. Second, this study was conducted in only three provinces, therefore, the results are not representative of Lao women in the whole country. Third, this study is a cross-sectional study, therefore, cause-effect relationships between PNC use and related factors cannot be established. However, this is the first study to examine the coverage of PNC 1–4 and factors associated with receiving two or more PNC services among rural Lao women. The findings of this study are useful for policy makers to develop strategies which promote continuum care for mothers and newborns.

## Conclusions

This study showed that the coverage of PNC 2 and 3 for mothers and newborns was low, while that of PNC 1 was high. Factors associated with having two or more PNC services for mothers and babies were being single/divorced, having a higher income, living outside the city, and having facility-based delivery. To increase the PNC utilization, institutional delivery with a minimum 24-hour stay after birth should be promoted. The Lao government should consider reintroducing targeted financial support for postpartum women, especially in low-income. Strengthening community outreach through local health workers and culturally appropriate education can further enhance access and uptake.

## Data Availability

The datasets used in this study are available from the corresponding author on reasonable request.

## Abbreviations

ANC: Antenatal care
AOR: Adjusted odds ratio
CI: Confidence interval
DHS: Demographic Health Survey
LAK: Lao Kip
Lao PDR: Lao People’s Democratic Republic
LMIC: Low- and middle-income country
MCH: Maternal and child health
MICS: The Multiple Indicator Cluster Survey
MMR: Maternal mortality ratio
NHI: National Health Insurance NHI
NMR: Neonatal mortality ratio
NGO: Non-governmental organization
OR: Odds ratio
PNC: Postnatal care
WHO: World Health Organization
